# Optimal Camera Pose and Placement Configuration for Maximum Field-of-View Video Stitching

**DOI:** 10.3390/s18072284

**Published:** 2018-07-14

**Authors:** Alex J. Watras, Jae-Jun Kim, Hewei Liu, Yu Hen Hu, Hongrui Jiang

**Affiliations:** 1 Department of Electrical and Computer Engineering, University of Wisconsin—Madison, 1415 Engineering Dr, Madison, WI 53706, USA; jkim724@wisc.edu (J.-J.K.); hliu265@wisc.edu (H.L.); yhhu@wisc.edu (Y.H.H.); hongrui@engr.wisc.edu (H.J.); 2 Department of Biomedical Engineering, University of Wisconsin—Madison,1550 Engineering Dr, Madison, WI 53706, USA; 3 Department of Ophthalmology and Visual Sciences, University of Wisconsin—Madison, 2828 Marshall Ct, Suite 200 Madison, WI 53706, USA

**Keywords:** optimal camera placement, sensor planning, image stitching

## Abstract

An optimal camera placement problem is investigated. The objective is to maximize the area of the field of view (FoV) of a stitched video obtained by stitching video streams from an array of cameras. The positions and poses of these cameras are restricted to a given set of selections. The camera array is designed to be placed inside the abdomen to support minimally invasive laparoscopic surgery. Hence, a few non-traditional requirements/constraints are imposed: Adjacent views are required to overlap to support image registration for seamless video stitching. The resulting effective FoV should be a contiguous region without any holes and should be a convex polygon. With these requirements, traditional camera placement algorithms cannot be directly applied to solve this problem. In this work, we show the complexity of this problem grows exponentially as a function of the problem size, and then present a greedy polynomial time heuristic solution that approximates well to the globally optimal solution. We present a new approach to directly evaluate the combined coverage area (area of FoV) as the union of a set of quadrilaterals. We also propose a graph-based approach to ensure the stitching requirement (overlap between adjacent views) is satisfied. We present a method to find a convex polygon with maximum area from a given polygon. Several design examples show that the proposed algorithm can achieve larger FoV area while using much less computing time.

## 1. Introduction

Camera arrays have extensive applications in surveillance [1], robotics [2], Virtual Reality [3,4,5], surgery [6], and more. Multiple cameras placed on proper locations and poses may offer a wider field of view (FOV) by aggregating (stitching) individual images into a coherent, extended mosaic beyond what a single camera can provide.

The traditional camera placement problem has been investigated in the context of video surveillance [7,8,9] where cameras are to be placed in a three-dimensional space to cover a two-dimensional plane [10]. Optimal camera placement under these restrictions has been previously investigated [11,12].

There are two types of camera placement problem formulations: MIN and FIX [9]. The goal of MIN formulation is to minimize the number of cameras needed to cover a given area. The goal of the FIX formulation is to maximize the coverage area for a fixed number of cameras. The work presented in this paper falls into the FIX formulation category.

**Figure 1:**
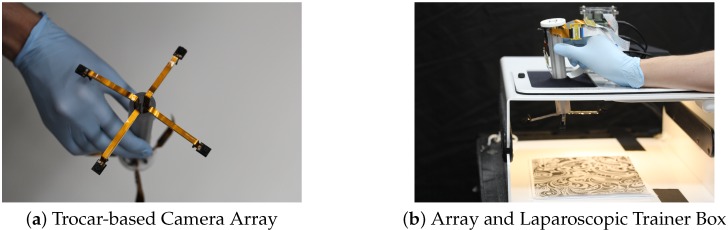
The trocar-based camera array offers improved FoV over traditional laparoscopes by utilizing multiple visual sensors. The extra field of view can encompass significantly more of the surgical area so that the surgeon does not have to spend as much effort adjusting camera location while performing surgical procedures.

The camera placement problem is formulated in the context of building a camera array to enhance the vision quality of a laparoscopic surgery [6]. In current laparoscopic surgery, a single-camera with lighting is inserted through a trocar to provide desired view for surgeons to carry out surgery. A camera array hanging on the trocar assembly will provide a bigger FoV while freeing the surgeon’s hand from holding the laparoscopic camera. The cameras will be mounted on four arms extending from the housing of a circular trocar, as shown in Figure 1. In this figure, the trocar assembly is mounted on a surgical training box to emulate the surgical setting.

To simplify the hardware design, the cameras will be fixed on pre-selected positions on each arm. At each position, the camera can be placed to have a specific pose selected from a fixed number of choices. The total number of cameras that can be mounted is also limited. This setup is able to enhance visual coverage during a laparoscopic surgery because FoVs from multiple cameras may be stitched together to provide a larger stitched FoV. Our task is to devise an algorithm to choose an optimal camera placement plan that maximizes the stitched FoV subject to several visual quality related constraints. These constraints make the camera placement problem formulation distinct from those in the existing literature.

The laparoscopic camera array is unique from existing work in that it is designed to provide a stitched FoV. Hence, a certain amount of overlap between adjacent views is required to facilitate image registration and sub-sequent stitching operation. View overlapping is not required for surveillance applications and hence has not previously been considered. In addition to view overlapping, we also require the stitched FoV to be contiguous and free of holes. To address the continuity requirements, we propose a graph based overlap checking method similar to the wireless network connectivity model [9]. To ensure that the resulting coverage region maximizes the usable working area for real time tasks and deals with blind spots, we adapt the potato peeling problem [13,14,15] to maximize the largest convex polygon that can fit within the coverage region rather than optimizing the full region. We also restrict allowable coverage regions to simple polygons as another means of removing blind spots (holes) from the coverage region.

The large number of camera positions that can arise from the combination of camera poses, positions, and types quickly makes an exhaustive search infeasible. However, The variability of the individual camera FoV means that most linear and nonlinear optimization methods cannot be used in this case. As such, we utilize a suboptimal greedy heuristic [16] to evaluate more complex camera positions.

Unlike previous works, the optimization method presented in this work includes a graph based method for ensuring that the global registration methods required for image stitching will still succeed. We also introduce a new objective function based on the Goodman et al. [13] “potato peeling problem” to remove blind spots that decrease the effective FoV for real time tasks such as surgery.

In Section 2 of this paper, we go over existing works on similar problems. In Section 3, we set up the model for stitching field of view and propose the optimization that stitching networks should attempt to solve. Section 4 formulates our solutions to the optimization problem to a surgical camera array which we have been developing. Section 5 shows the results of the optimization in several cases. Finally, Section 6 discusses the results of the various approaches to the optimization problem.

## 2. Related Works

One of the earliest surveillance coverage problem formulation is the Art Gallery Problem (AGP) [17]. The goal here is to minimize the number of visual sensors (cameras) required to monitor the floor plan of an art gallery (region of interests, ROI). However, the sensor model in the AGP expected sensors to see any object within line of sight of the sensor and limited the camera and surveillance regions to a two-dimensional space. Many existing solutions of the AGP problem do not work well for camera sensors with specific directionality properties. As such, the AGP needs to be reformulated to better reflect real camera models and spatial placement constraints [7,8,9].

Some AGP-derived problem formulations attempt to use a two-dimensional floor plan and two-dimensional camera models [9,18,19,20,21,22]. These models benefitted from their simplicity as camera FoV could be treated as a simple, static two-dimensional shape which enabled many optimization techniques. However, they relied heavily on assumptions about camera placement and scene shape. As such, their usefulness is limited and not applicable to the surgical setting considered in this work.

Several works expand the two-dimensional world view of the AGP into a more realistic three-dimensional case [7,16,20,23,24,25,26,27,28]. However, while many of these models can be simplified when you care about a planar scene, these models require extra computation to be used for occlusion checking which is unnecessary when dealing with a planar scene. These methods typically revolve around discretizing the scene space into a grid and seeking to maximize the number of grid points visible to the camera array. This scene discretization leads to a source of possible error that is not necessary when the scene is planar.

The restriction of 3D scene geometry to a two-dimensional plane has been previously proposed for the purposes of maximizing coverage of a floor plan by surveillance equipment [11,12,29] . Fu et al. [11] proposed a two-dimensional coverage model for the placement of cameras in three-dimensional space that is very similar to ours. They used a particle swarm optimization method to perform an optimization that attempts to simultaneously optimize for both maximum coverage of the surveillance space and minimum number of used cameras. However, they did not apply the greedy heuristic, look at continuity constraints, or look at ensuring overlap as we need to for image stitching. Piciarelli et al. [12] also looked at placing camera sensors in three-dimensional spaces to record scenes on a two-dimensional plane. However, their camera model does not approximate the real world behavior of a camera as well as the model used by Fu et al. [11].

## 3. Methods

### 3.1. Problem Formulation

We formulate an optimization problem to attempt to maximize the FoV of our camera array while ensuring that the chosen array will still create a useable stitched mosaic. To do this, we need to mix image stitching constraints with optimal camera placement models.

Similar to the traditional models [7,8,9], we discretize the camera positions based on our array structure. Our choice of camera position constraints will depend on where the camera network is meant to be deployed. For this paper, we largely focus on a set of three different possible sets of constraints intended for use in our surgical camera array, which we naive, symmetric, and asymmetric camera placement.

We then evaluate a coverage model similar to the one used by Fu et al. [11]. While this model often uses additional constraints [8,11] such as resolution and focus which serve to limit the camera FoV even further, these bounds are unnecessary in close field applications such as our desired surgical application.

We model our scene as a plane in space as in Fu et al. [11]. This follows naturally from the requirements of stitching that the recorded scene be approximately planar [10]. The planar scene constraint also precludes any need for occlusion handling as occlusion will not occur in a purely planar scene. Thus, occlusion handling methods [8] are not included in the model. In this setting, we utilize a plane which represents our approximation of the image stitching plane.

If we know the coordinates of this plane exactly, then we can simply utilize the camera projection model to generate the image mosaic. The resulting mosaic is not robust to noise and will cause misalignment if the planar scene is not exactly as expected. As such, even when we expect to know the scene well, we still desire to perform traditional image stitching in order to minimize the likelihood of misalignment.

Image stitching requires sufficient overlap between adjacent cameras to find the feature matches used to compute image correspondence. For multi-camera image stitching, each camera must be able to trace a path back to the main-view camera, with each step along the path transitioning between two cameras which share sufficient overlap. To ensure this continuity constraint is figured, we propose a graph based method for checking if a global image correspondence can be achieved.

Rather than discretize the scene space as in many previous works, we instead choose to evaluate the area of the continuous coverage region on the two-dimensional plane in order to more accurately evaluate the total coverage. Since each camera’s coverage region is the intersection of a rectangular pyramid with the plane of stitching, it is easy to see that each camera will contribute a single quadrilateral to the overall coverage region. The area of the resulting coverage region can be quantified as simply the area of a union of quadrilaterals which makes it simple to compute.

After determining how to calculate the area of scene which is visible from a given camera set up, we propose the following optimization. Given a stitching plane, a number of known cameras which can be placed in a discrete set of poses and locations, and a minimum threshold for overlap between the camera views to allow for image stitching, the cameras should be placed in such a way that:

(1) $$\max_{R_{i},{\bar{t}}_{i}}A\left(\cup_{i}Q_{i}\right)$$

(2) $$Q_{i}={\left[B_{i1},B_{i2},B_{i3},B_{i4}\right]}^{T}$$

(3) $$B_{ij}=\lambda_{ij}R_{i}V_{j}d_{i}+{\bar{t}}_{i}$$

(4) $$\lambda_{ij}=\frac{{\bar{t}}_{i}{\left[\nu ,\tau ,\psi \right]}^{T}+\eta }{R_{i}V_{j}d_{i}{\left[\nu ,\tau ,\psi \right]}^{T}}$$

(5) $$g(\cup Q_{i})=1$$

where A(Q) is the function for the area of the polygon *Q*. $Q_{i}$ denotes the quadrilateral defined by the intersection of camera *i*’s viewing cone with the plane of stitching. Index $i=\{1,2,\ldots ,N_{c}\}$ refers to which camera we are using, and index j={1,2,3,4} refers to the corners of the viewing cone for that camera.

**Figure 2:**
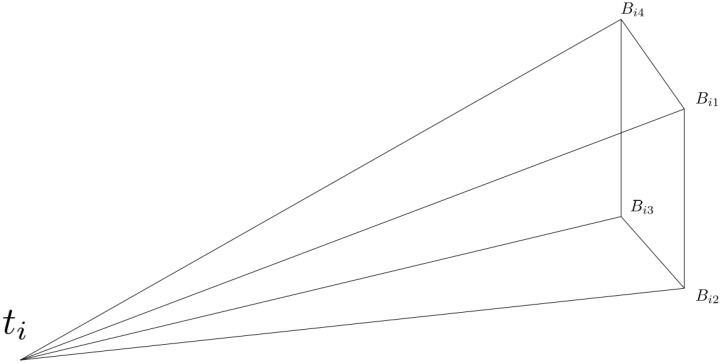
The camera viewing cone is defined by it’s four corner vectors ($B_{i}j$) and the camera center ($t_{i}$).

The viewing cone (as shown in Figure 2) is defined by the camera center and four vectors denoting the four corners of the viewable region. These vectors are described in Equation (3), where $R_{i}$ and ${\bar{t}}_{i}$ denote the camera *i*’s rotation and translation matrices, $d_{i}$ contains the information about the maximum viewing angles for camera *i*, and $V_{j}$ is a matrix that selects the vector corresponding to the *j*th corner of the viewing cone for that camera.

The stitching plane coefficients [ν,τ,ψ,η] define the stitching plane with νx+τy+ψz+η=0 being the plane of stitching and $\lambda_{ij}$ determining the length of $B_{ij}$ when projected onto the stitching plane using ray-plane intersection as in Equation (4).

**Figure 3:**
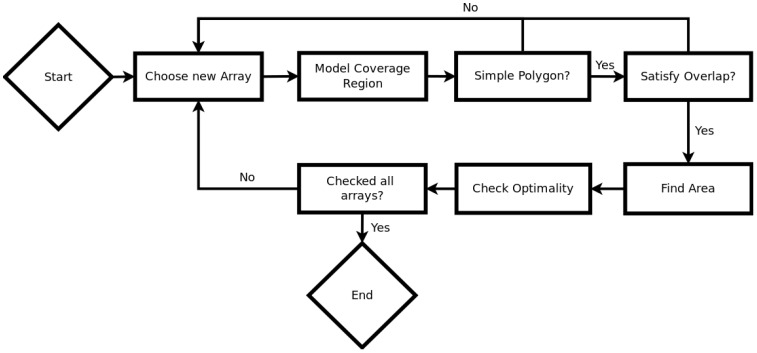
The general flow of our optimization algorithm. For each possible array, we model the coverage region, check to see that the result is a simple polygon which satisfies the overlap constraints, then find the area and compare that to the previously maximum area array.

Since we are dealing with a discrete set of possible camera poses and locations, A(Q) can be computed prior to run-time and stored so that it can simply be accessed from a look up table when checked for each possible camera set up. Thus, it will scale linearly with the number of allowable camera poses, but not scale up with the total number of array set ups. g(Q) counts the number of simple polygons required to describe the polygon *Q*. This serves to simplify computation of the area of the final FoV and it ensures that the final FoV does not have any holes. This is relevant to most applications since a hole usually represents an area close to the region of interest for which we do not have data. In surveillance, a hole could be exploited to hide information from the cameras, and, in surgical settings, a hole could cause the surgeon to miss out on information about tissues or organs near their surgical tools. In some settings, where the region of interest is oddly shaped, holes may be allowable, however, in many, it is not. To enforce these two additional constraints, we follow the algorithm outlined in Figure 3.

$R_{i}$ and ${\bar{t}}_{i}$ are the rotation and translation matrices for each camera. Our optimization is over all possible sets of rotation and translation available to our cameras. The construction of the array itself limits the possible camera poses, and acts as additional constraints on our problem. Without constraining camera pose, the problem is ill defined. However, the camera pose constraints are heavily dependent on the design of the array itself.

Unfortunately, this method requires checking each possible array set up to determine which solution is optimal if we consider a camera array where each camera can move and rotate freely. This creates six degrees of freedom for the camera (three degrees of rotation, and three degrees of translation). When we discretize the camera positions, if we allow *n* discrete values along each degree of freedom, this would cause there to be $n^{6}$ possible placements per camera. Thus, a *k* camera array would have approximately $n^{6k}$ possible arrangements. In reality, we can condense the number of arrangements slightly. No two cameras can be placed in the same position though they are allowed to share the same rotational pose. This means that we can have at most n3k possible positions rather than $n^{3}k$. Thus, the total magnitude of the solution space is actually n3kn3k.

As the number of allowable camera positions grows, solving our optimization quickly becomes computationally infeasible. To speed up computation time, we utilize a greedy heuristic to vastly simplify the computation time. While this heuristic is not guaranteed to find an optimal solution, It still provides good results, as seen in the paper by Zhao et al., and can simplify the exhaustive search algorithm down to polynomial time [9].

### 3.2. Scene Space Model

Most existing works in optimal camera placement utilize a discrete scene space model. In this model, rather than calculate the exact coverage region, a discreet grid of scene points is overlaid over the scene. The optimization function will then seek to maximize the number of these scene points which are covered by the camera array. This model helps to simplify computational constraints of the camera model and objective functions at the cost of some amount of accuracy in the size of the coverage region.

One of the largest benefits of using a discrete scene space model is that it allows the use of binary integer programming techniques that would not otherwise be possible. However, these techniques also require that the system constraints be expressed as a linear function. Since the computation of the individual camera coverage is a nonlinear function, the binary variable $b_{ij}$ which denotes whether a discrete scene point *j* can be seen from a camera placed at position *i* must be computed and stored in full before the optimization can be performed. As such, as the camera coverage model becomes easier to evaluate, the the binary optimization model becomes less preferable.

Without occlusion handling required in our setting, computing our individual camera coverage model is simple, although it is nonlinear and our constraints mean that evaluating the coverage region is a simple matter of performing a union of quadrilaterals using polygon clipping techniques [30] and then calculating the area of the resulting simple polygon. Using a continuous scene space allows us greater accuracy for the simple exhaustive search methods we wish to use to evaluate our camera array’s coverage quality.

### 3.3. Ensuring Continuity

To allow for stitching, we need to ensure that there is enough overlap between the cameras for feature matching to occur. The general approach to stitching together video from camera arrays is to find pairwise homographies which will transform the images such that features are matched between the resulting images. The amount of overlap required will vary based on the feature density of the scene viewed. However, assuming we know the amount of overlap required between our images for our expected scenes, we can set the following limitations on our set up to ensure that stitching may occur. We define the amount of overlap between two cameras to be the area of the intersection of their fields of view. To form a panorama from the cameras, we need to ensure that we can reasonably determine where each image needs to be in respect to all of the others. To do this, we propose a graph based method similar to the network connectivity method used by wireless sensor networks [9]. To generate our graph, we use the following steps:

1. Let each camera be a node in the graph.
2. Let the weight of an edge of the graph be the area of the intersection of the two connected nodes of the graph.
3. Apply a simple threshold to remove any edges of the graph which do not satisfy the requirements for stitching.

**Figure 4:**
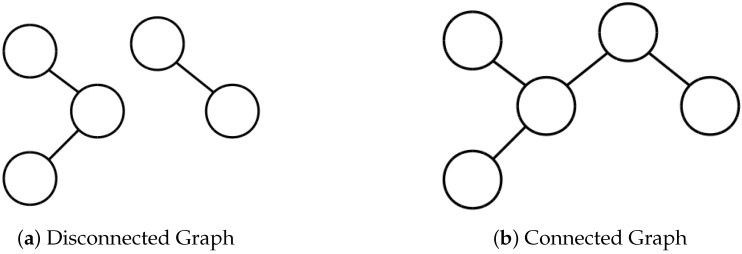
To determine whether stitching is possible, we need to check that there is sufficient overlap between the views. To do this, we create a graph where each node represents a camera and the existence of an edge specifies that there is enough overlap between the two cameras that stitching could occur. If the resulting graph is connected, then we will be able to create a mosaic from the camera array.

If the resulting graph is connected as in Figure 4b, this tells us that, from any given camera, we can create a path such that we reach every other camera and all of the paths transversed have an overlap greater than our desired threshold. This is equivalent to saying that we can connect any image from a camera in our array to any other camera image by chaining together feature matches. This allows pairwise stitching together all of the cameras. However, if the graph is disconnected as in Figure 4a, then our camera array covers two disconnected scenes and we have no way of understanding how those two scenes should interact.

The threshold chosen represents how much overlap is needed to find sufficient feature matches between cameras to perform stitching. In general, there is a minimal number of feature matches required to compute the proper transformations required for image stitching. Therefore, it would make sense that our threshold could be chosen based on the expected density of features in the scene so that we could try to ensure that minimum number of feature matches is fulfilled. However, the method outlined in Section 3.2 already gives us the relationship between the camera views from simply the camera pose and the stitching plane. Thus, if we know the stitching plane exactly, this threshold can be 0 and we can simply ensure that our resulting scene is continuous without needing any overlapping feature points. As our uncertainty about our estimated stitching plane grows, so to does the need for a high threshold to ensure that we can perform stitching through traditional means.

The adjacency matrix *C* of the camera FoV $Q_{i}$ can be defined as the $N_{c}\times N_{c}$ matrix with

$$C_{ij}=\left\{\begin{array}{cc} 1 & A(Q_{i}\cap Q_{j})>\tau \\ 0 & A(Q_{i}\cap Q_{j})\leq \tau \end{array}\right.$$

Using this adjacency, we can check whether *C* is connected by checking if the matric $C^{\prime }=\sum_{k=0}^{n}C^{k}$ has any nonzero elements. Therefore, the block of constraints added to our optimization by the connectivity constraints are:

(6) $$C_{ij}=\left\{\begin{array}{cc} 1 & A(Q_{i}\cap Q_{j})>\tau \\ 0 & A(Q_{i}\cap Q_{j})\leq \tau \end{array}\right.$$

(7) $$C_{ij}^{\prime }=\sum_{k=0}^{n}C_{ij}^{k}$$

(8) $$\sum_{i=1}^{N_{c}}\sum_{j=1}^{N_{c}}\left|C_{ij}^{\prime }\right|\leq 0$$

### 3.4. Blind Spots

**Figure 5:**
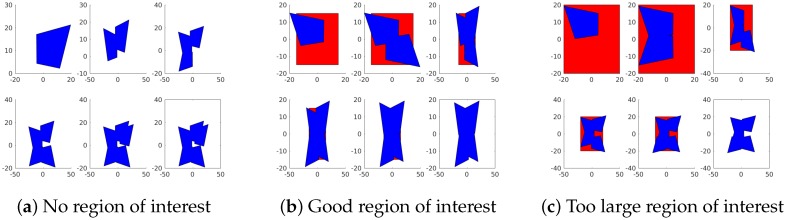
The placement of cameras in the array following the greedy algorithm for various regions of interest. Blue shapes are the total coverage of cameras in the array, and red indicates the region of interest used. The plot in the lower right of each figure shows the resulting field of view without the region of interest.

One of the potential dangers of optimizing camera placement for maximal FoV is that the viewable region that results may be irregularly shaped, as shown in Figure 5c. While these figures may offer the largest total FoV, they may not be practical for many applications due to the portions of the scene that are omitted from the region. If these regions contain important information about the scene, then the effective gain in FoV over other camera configurations may be significantly lessened.

While it can be difficult to quantify the effect that coverage irregularity has on effective FoV, there are some approaches that can be used to reduce the amount of coverage irregularity that occurs. While the evaluation and comparison of these approaches is left out as it is beyond the scope of this paper, their potential is still worth noting.

First, A region of interest (RoI) can be used to try and focus the FoV on the portions of the scene that need to be covered. Rather than simply maximizing the total size of the coverage region, we instead define a space that we wish to cover and maximize our coverage of that region. This method is sensitive to the choice of RoI. As can be seen in Figure 5, different choices of a region of interest can result in very different coverage regions and having a region that is too large or poorly shaped to be covered by the array given camera placement constraints can be just as bad as having no region of interest.

Another approach is to change the cost function for the optimization to better reflect our desire to create as large a region without blind spots as possible. A region without blind spots can be thought of as a region *P* such that, for any two points, x,y∈P. The direct path is $\bar{xy}\subseteq P$. We can see that a region without blind spots is equivalent to a convex region. Thus, rather than maximizing the area of the FoV, we can instead seek to maximize the largest convex region fully contained within the FoV. Finding the largest convex region inside of a non-convex polygon was originally proposed and dubbed the “potato peeling problem” by Goodman [13] and the “convex skull problem” by Woo [14] and was later solved in polynomial time by Chang and Yap [15]. Other works have found solutions or approximate solutions to sub-problems such as the largest inscribed rectangle [31,32], longest line segment, or largest ellipse [33] contained within a non-convex polygon.

The largest rectangle problem is of particular interest to camera arrays as cropping the resulting mosaic into a rectangle would cause the camera array to behave more similarly to a traditional single camera set up. However, for the purposes of this paper, we decided that cropping the mosaic down to the largest inscribed rectangle would disregard too much of the information received by the array.

The method proposed by Chang and Yap solves the potato peeling problem in $O(n^{6})$ time. We instead chose to use a method that approximates the solution in $O(n^{2})$ time since we need to solve the potato peeling problem for every proposed solution in our discretized solution space. We utilize the Butterfly Lemma provided and the resulting linear time solution when the non-convex polygon has only one reflex corner. By applying this solution to each reflex corner of our non-convex polygon, we generate a series of cuts, each of which is chosen such that it removes as little area from the polygon as possible. By applying each of the cuts to the polygon, we can then generate an approximate solution to the potato peeling problem. This approximation can fail to find the correct solutions when the optimal solution involves chains of butterflies with length more than 1, but generates good solutions in many cases.

For our optimization, we simply create a function p(Q) which peels the polygon *Q* into the largest convex polygon $Q^{\prime }\subset Q$. Rather than maximizing A(Q), we now seek to maximize $A\left(Q^{\prime }\right)=A\left(p\left(Q\right)\right)$.

### 3.5. Greedy Heuristic

When we allow more freedom for camera placement, the complexity of the problem quickly makes exhaustive search infeasible. Thus, to improve performance of camera arrays with high amounts of freedom in pose and position, we propose a greedy suboptimal algorithm similar to the one proposed by Horster et al. [16].

In addition, since it is typically best to maximize the angle of the camera relative to the stitching plane, we may miss sections of the scene close to the center in favor of distant portions of the scene where the cameras can cover a lot of area. To ensure that we cover everything important about the scene, we introduce the concept of a region of interest.

We consider the case where we have a region of interest that we wish to cover with the camera array. Our goal now becomes to cover the region of interest while still maximizing the total area that we can see. We are still limited by the stitching constraints, namely that our scene is planar, and that we require a minimum amount of overlap between the cameras so that a sufficient number of feature matches can be gathered. This is similar to the polygon covering problem which is NP-hard [34,35].

The methods described earlier in the paper allow us to determine the total field of view of an array of cameras given their position. Therefore, we attempt to build a camera array which will primarily maximize the coverage of the region of interest and secondarily maximize the total field of view.

The greedy algorithm we propose is as follows:

1. Compute the footprint of all possible poses for the camera.
2. If cameras have been placed already, identify the region of overlap with all previously placed cameras and discard all poses which do not overlap with the previously placed cameras.
3. Identify the region of overlap between each pose and the region of interest.
4. Choose the pose for which this region has maximum area.
5. If two or more poses are tied, choose the pose whose footprint has the maximum area.
6. Remove the chosen footprint from the region of interest.
7. Repeat steps 1–6 for each other camera in your array.

**Figure 6:**
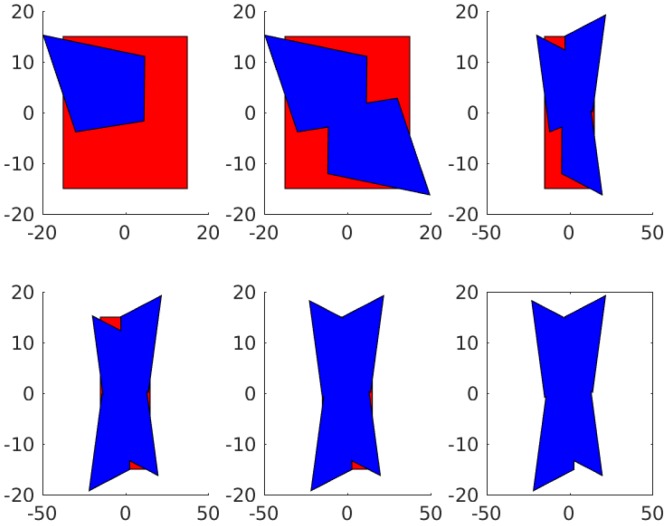
The placement of cameras for the greedy algorithm. The blue region denotes what area the camera can see, and the red region denotes the region of interest we wish to cover.

When applied to a sample set of restrictions for a grid based camera array, the resulting field of view for the array after the placement for each camera can be seen in Figure 6.

If we consider a discretization model for a camera array of *k* cameras that allows each camera to be placed in *n* different locations, in one of $N_{p}$ different poses. We can see that using this algorithm, We need to check all $N_{p}$ poses for each camera, but for the *i*th camera we need to check only n−i+1 locations to find our choice of solution. Thus, our resulting solution requires simply $\sum_{i=0}^{k-1}N_{p}\left(n-i\right)\leq N_{p}nk$ evaluations of the field of view of an array. Thus, our suboptimal solution is only O(n), whereas the exhaustive optimal solution is $O(a^{3}n)$. While this method is not guaranteed to find the optimal solution, it will allow for the computation of a suboptimal solution in a linear time rather than exponential.

### 3.6. Unified FIX Optimization

Including all of our additional connectivity and convexity constraints, we can re-write our optimization as

(9) $$\max_{R_{i},{\bar{t}}_{i}}A\left(p\left(\cup_{i}Q_{i}\right)\right)$$

(10) $$g(\cup Q_{i})=1$$

(11) $$Q_{i}={\left[B_{i1},B_{i2},B_{i3},B_{i4}\right]}^{T}$$

(12) $$B_{ij}=\lambda_{ij}R_{i}V_{j}d_{i}+{\bar{t}}_{i}$$

(13) $$\lambda_{ij}=\frac{{\bar{t}}_{i}{\left[\nu ,\tau ,\psi \right]}^{T}+\eta }{R_{i}V_{j}d_{i}{\left[\nu ,\tau ,\psi \right]}^{T}}$$

Using this framework, we can either use an extensive search method or our proposed greedy heuristic to evaluate the quality of allowable camera poses and choose an optimal camera array for a given camera space. The resulting camera array should be optimal (or near optimal in the case of the greedy heuristic) over all camera arrays found in the camera space.

## 4. Camera Spaces

As we can see in Figure 1, the trocar camera array on which we performed the optimization heavily limited in translation and rotation. We have cameras placed along four telescoping arms. The cameras can be angled towards or away from the center of the array.

These constrictions mean that each camera has three degrees of freedom. Let $\gamma \in \{-\frac{\pi }{2},0,\frac{\pi }{2},\pi \}$ denote the rotation due to the choice of arm, $t\in [t_{min},t_{max}]$ denote the position along that arm, and $\theta \in [\theta_{min},\theta_{max}]$ be the rotation towards the center of the array. Then, the triple $Cam_{i}=\left[\gamma_{i},t_{i},\theta_{i}\right]$ defines a camera position for camera *i*.

From these parameters, the camera translation and rotation matrices should be defined as follows.

Rotation matrices can be created from their individual components in each of the axes of the computational space

$$R_{x}\left(\alpha \right)=\left[\begin{array}{ccc} 1 & 0 & 0\\ 0 & \cos(\alpha ) & -\sin(\alpha )\\ 0 & \sin(\alpha ) & \cos(\alpha ) \end{array}\right]$$

$$R_{y}\left(\beta \right)=\left[\begin{array}{ccc} \cos(\beta ) & 0 & \sin(\beta )\\ 0 & 1 & 0\\ -\sin(\beta ) & 0 & \cos(\beta ) \end{array}\right]$$

$$R_{z}\left(\gamma \right)=\left[\begin{array}{ccc} \cos(\gamma ) & -\sin(\gamma ) & 0\\ \sin(\gamma ) & \cos(\gamma ) & 0\\ 0 & 0 & 1 \end{array}\right]$$

Then, the final rotation matrix can be computed by re-combining the individual matrices.

(14) $$R=R_{x}\left(\alpha \right)*R_{y}\left(\beta \right)*R_{z}\left(\gamma \right)$$

In our case, α=θcos(γ), β=θcos(γ).

The translation matrix that should be applied to each camera should be:

$${\bar{t}}_{i}=\left[\begin{array}{c} t_{i}sin\left(\gamma_{i}\right)\\ t_{i}cos\left(\gamma_{i}\right)\\ 0 \end{array}\right]$$

### 4.1. Asymmetric Camera Space

In our asymmetric camera space, we allow the cameras to be placed in any position and pose allowable by the construction of the camera array. For the trocar array in Figure 1, this means that the $Cam_{i}$ has the full three degrees of freedom γ,t,θ. Cameras are only limited by the fact that no two cameras may share the same [γ,t] pair as no two cameras may be in the same space and the individual limits on gamma,t, and θ that are imposed by the camera space discretization. Using the asymmetric camera space allows us the most freedom for our camera placement, but with $O(3^{n})$ degrees of freedom it also has the largest solution space which means it limits the allowable discretizations of the camera space.

### 4.2. Symmetric Camera Space

In this case, assume that the array is restricted to placing four of the cameras at the same arm length and angle, with a fifth camera placed at the center of the array to serve as the main reference viewpoint for stitching. In this case, we control the angle of all the cameras θ, and the arm length *l*. This adds the additional restriction that

(15) $$\theta =\theta_{1}=\theta_{2}=\theta_{3}=\theta_{4}$$

(16) $$t=t_{1}=t_{2}=t_{3}=t_{4}$$

Each of these cameras is placed on its own arm, so

$$\gamma_{1}=-\frac{\pi }{2},\gamma_{2}=0,\gamma_{3}=\frac{\pi }{2},\gamma_{4}=\pi$$

Our fifth camera, we place at the center of the array with $t_{5}=0$, $\theta_{5}=0$, and $\gamma_{5}=0$.

**Figure 7:**
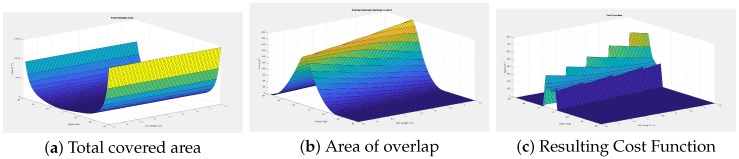
Applying the model to a set of five cameras placed symmetrically on the trocar camera array shows the following: (**a**) the area of the union of the fields of view; (**b**) the area of overlap between Cameras 1 and 2; and (**c**) the cost function that results from thresholding the overlap between each pair of cameras.

This allows us to simplify the computation to optimization over just two parameters [t,θ] and can be visualized easily since it has so few degrees of freedoms. The resulting cost function when applied to our surgical set up can be seen in Figure 7.

From these figures, we can see that camera angle appears to be the major driving factor for total area and that maximal coverage appears where angle is maximized and overlap is minimized. This seems reasonable as the FOV of an individual camera increases as the camera is rotated relative to the stitching plane, and the total field of view is simply the union of the individual cameras.

### 4.3. Naive Camera Space

**Figure 8:**
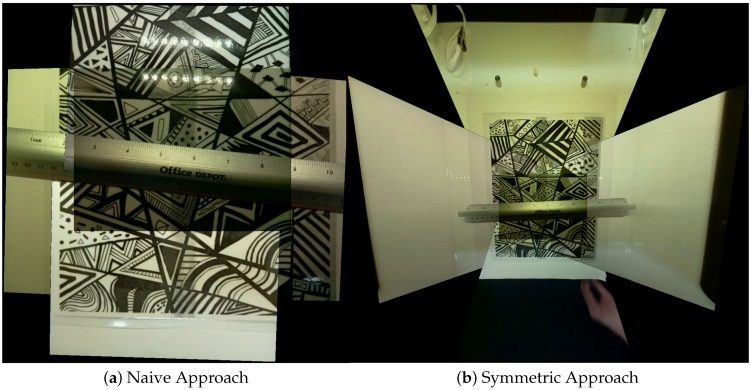
Applying symmetric optimization to create camera arrays for use in a laparoscopic trainer box can give significant improvements in total visibility for tasks in the box: (**a**) a frame from the original naive camera system; and (**b**) a similar frame from our symmetrically optimized camera system. The optimized system gives us near complete coverage of the test box. No blending techniques were applied so that the individual camera views can be easily picked out from the mosaic.

In the naive camera space, we simplify even further down to the single parameter *t*. The naive camera space represents the approach that one might initially think of for optimizing the trocar camera array. All cameras are placed facing in the same direction as the main reference camera and then moved to create the maximum allowable spread of the cameras. However, we can see that limiting ourselves to a single degree of freedom sacrifices a significant amount of possible coverage. Figure 8 shows the improvement in field of view for our laparoscopic test box in using the symmetric approach rather than the naive approach.

## 5. Results

**Figure 9:**
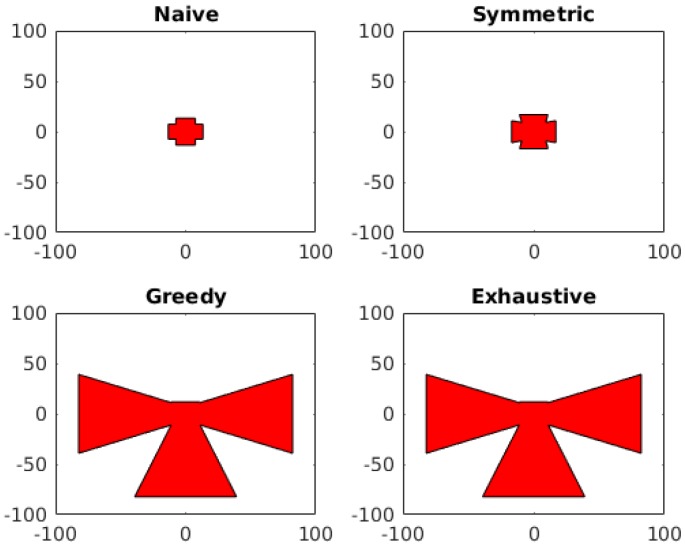
The resulting views of the four approaches applied to the restrictions of the trocar camera array.

The model optimization was run under the constraints required by our image stitching system. The plane of stitching was chosen to be the plane z=16.5 to simulate a camera array placed at the ceiling of a laparoscopic trainer box with the camera array pointed directly at the surgical area. Due to the computational restrictions in computing the result in the exhaustive case, we must limit our space of possible camera poses to a relatively small number. We chose to create an array of five cameras using our trocar based array frame. Each camera was allowed to be placed on one of the four arms of the frame at one of two possible positions on the arm. The camera was then allowed to be rotated into one of three possible poses. Since we cannot have two cameras in the exact same position on the camera arms, this gives us 8535 = 13,608 possible configurations to check in the exhaustive case. The resulting fields of view from this test set up can be seen in Figure 9 and the total areas corresponding to each method are shown in Table 1.

The upper bound provided is a loose upper bound determined based on the maximum field of view of a single camera. Let us define $A_{i}$ as the field of view area of a camera at position/pose index i=1,2,3…,n, *k* as the number of cameras to be placed in the array, and τ as the minimum amount of overlap such that we meet our overlap restrictions from Section 3D. Then, the upper bound *U* is defined as

$$U=\max_{i}kA_{i}-\tau$$

While this upper bound does not give us an achievable bound that our algorithm could approach, it serves to give us an idea of the result we would get if we could somehow find a way to set up the cameras such that each was individually maximized and they still had the minimum amount of overlap. It is important to note that even the exhaustive case does not manage to approach the upper bound due to the fact that the cameras must be placed in our array and the restrictions due to the discretization of the problem.

Using this upper bound, we can get a feel for how well our greedy algorithm can perform in cases where the total computational complexity for the exhaustive method would be much too high to compute. For example, we consider placing the cameras on a two-dimensional grid such as in the Stanford camera array [36], allowing for rotation around both the x and y axes. For this camera case, our naive approach allows for a camera to be placed at any spot in the array as long as no rotation is applied to it, and our symmetric approach places the cameras on the grid in the same pattern that they would have been on the trocar array with the cameras still arrayed in the cross pattern of the trocar arms and still required to rotate towards or away from the center.

**Figure 10:**
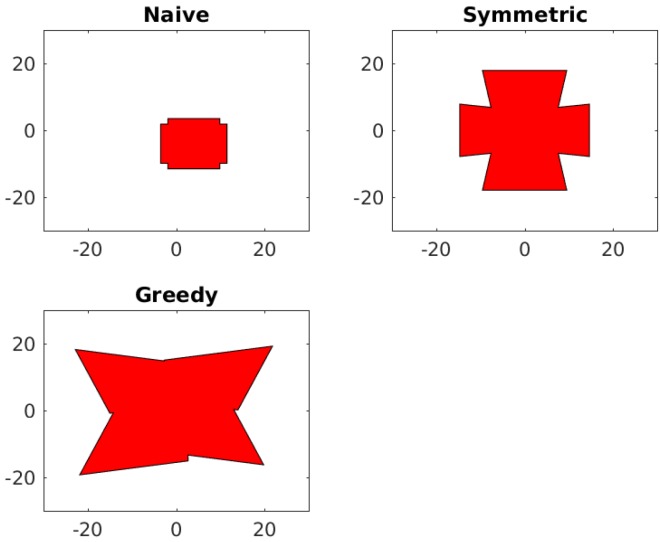
The resulting field of view of the three approaches used for the grid based camera array.

The results of this optimization can be seen in Table 2, and the corresponding fields of view can be seen in Figure 10.

**Figure 11:**
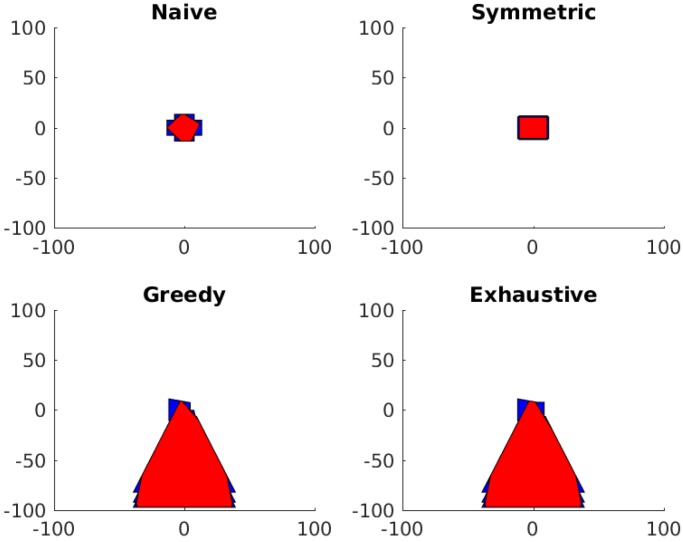
The resulting field of view when optimizing for maximal convex region rather than maximum total area. Blue represents the camera FoV and red represents the found convex region.

Next, the constrained system for the surgical camera array is evaluated for the maximal convex region rather than the maximal area region. The resulting regions can be seen in Figure 11 and the resultant area comparison can be seen in Table 3.

## 6. Discussion

While the maximal area approach unsurprisingly achieved a larger area than the maximal convex region approach and the resulting coverage region is nicely symmetrical, as we can see in Figure 5, removing the region of interest can cause the construction of the array to become very irregular and it would not be very useful for most mosaicking applications. However, even a poorly matched region of interest does a good job making an array which has a much larger field of view than the naive approach. However, the array coverage may be deceptively large as the result consists of three major regions that are only connected to one another by a very small region.

The maximal convex region approach is able to generate a region which is much more similar to what could be seen by a single large sensor as we desire for image stitching. The cost to the size of the array appears to be very large in this particular case, however, with most of the coverage region being dominated by a single camera and the other sensors not contributing nearly as much to the region. This likely follows from our restrictions on camera placement which cause a single camera with a high level of rotation to achieve a much higher total field of view than a large group of cameras placed so that they fulfill the convexity requirements since there are significantly fewer placements which can create large convex regions.

In addition, it is important to pay attention to restricting camera angle. Since we define our field of view as the intersection of the FoV cone with the stitching plane, if we allow for unrestricted rotation, we can easily allow for infinite FoV with a single camera by placing our camera with a high enough angle relative to the field of view. However, these higher angled poses also lead to mosaics that do not feel like they accurately model the real world, as we can see in the slight tunnel vision effect that occurs in Figure 8 so restricting angle is necessary for good looking mosaics. We could discourage these high rotation cases by simply disallowing them from our array construction, by re-adding the resolution constraints from Mavrinac et al. [8], and/or by our choice of region of interest. Even with the resolution constraints, we require our cameras to be placed further away from the actual scene when using high rotation cameras without a region of interest, so a mixture of the proposed methods in this paper may be preferable.

## 7. Conclusions

In this paper, we have proposed a new optimization scheme for the purposes of image and video stitching. We leverage the constraints of stitching to propose a set of constraints on camera placement and view. Using those constraints, we propose a FIX optimization problem for placing a set number of cameras to create maximal FoV.

Since the FoV of an individual camera on our scene exhibits nonlinear properties, we cannot use linear programming methods to perform the optimization. Instead, we discretize the solution space into a finite discrete set of positions and poses. To ensure accuracy of the stitched image if our estimate for the stitching plane is incorrect, we additionally constrain our solution space such that there must be sufficiently large overlap area between images captured by a pair of cameras. The amount of overlap required will depend on the feature density of the scene, thus this is left as a tuneable parameter depending on the scene.

We formulate this problem as a constrained discrete optimization problem and show the solution space grows exponentially as the number of camera grows. A sub-optimal, greedy heuristic for solving this problem in polynomial time is presented to allow the extension of this problem to less heavily constrained arrays. The greedy algorithm is not intended to be an optimal solution to the optimization problem proposed in the paper. Instead, it is intended to be a method for evaluating the optimization model for complex camera arrays and improve camera array placement over the naive approach that can be computed quickly even when run on a large space of possible camera placements. To the best of our knowledge, this is the first such work that seeks to maximize the field of view of the stitched image subject to the overlapping constraint required for proper stitching.

To do so, we needed to introduce a continuity constraint to ensure that cameras had sufficient overlap and a continuous path of camera overlap so that stitching could be performed. In addition, there is a requirement that our coverage region be free of holes, since many optimal solutions that do have holes in them do not make for good mosaics.

To further tailor our optimization to the needs of the stitching setting, we propose a new measure for maximal FoV that better reflects the stitching desire to create a large region that mimics the behavior of a single visual sensor. Rather than focusing on the total area of the coverage region, we instead focused on a subset of the coverage region. First, we considered a region of interest based approach, but we discovered that the behavior of this approach is very heavily dependent on the chosen region of interest, with size variations in the region leading to some potentially unusable camera arrays. Next, we tried a variant of the potato peeling algorithm which seeks to find the largest convex region that fits inside our FoV. This seems to behave more as desired, but does not take into account that some regions may be more desirable to monitor than others. However, these two approaches give the user more ways to control how they wish to place cameras to monitor a scene.

For future work in this area, we are interested in developing a MIN optimization for image stitching. We are also interested in investigating how these models can be used to improve the construction of camera arrays when 3D reconstruction is the goal rather than image stitching and looking at whether the same model can be applied to light generation sources such as lighting for theatrical performances and art installations rather than light collection sources as in this paper.

## Figures and Tables

**Table 1:** Results of Maximal Area Optimization Methods on Surgical Array.

Approach	FOV Area (cm^2^)	Evaluation Time (s)
Naive	578	0.122
Symmetric	955	0.177
Greedy	11,220	0.519
Exhaustive	11,220	38,274
Upper Bound	18,214	0.073

**Table 2:** Results of Maximal Area Optimization on Grid Array.

Approach	FOV Area (cm^2^)	Evaluation Time (s)
Naive	215	1.423
Symmetric	795	0.700
Greedy	1153	15.862
Upper Bound	1514	0.203

**Table 3:** Results of Maximal Convex Region Optimization on Surgical Array.

Approach	Max Area (cm^2^)	Max Conv. Reg. (cm^2^)
Naive	577	577
Symmetric	795	795
Greedy	11,220	5069
Exhaustive	11,220	5104
